# Varicella zoster-associated retinal and central nervous system vasculitis in a patient with multiple sclerosis treated with natalizumab

**DOI:** 10.1186/1742-2094-11-19

**Published:** 2014-01-30

**Authors:** Xenia Kobeleva, Florian Wegner, Inez Brunotte, Mete Dadak, Reinhard Dengler, Martin Stangel

**Affiliations:** 1 Department of Neuroradiology, Medical School Hannover, Carl-Neuberg-Straße 1, 30625 Hannover, Germany; 2 Department of Ophthalmology, Medical School Hannover, Carl-Neuberg-Straße 1, 30625 Hannover, Germany

**Keywords:** Multiple sclerosis, Varicella zoster virus, Vasculitis, Natalizumab

## Abstract

We report the first case of combined retinal and CNS varicella zoster-associated vasculitis in a 49-year-old patient with multiple sclerosis who had been treated with natalizumab. He presented with a progressive bilateral visual loss. The diagnosis of a vasculitis was based on the fundoscopic examination and MRI findings. We confirmed the varicella zoster virus (VZV) infection of the CNS by PCR and increased intrathecal antibody indices in the cerebrospinal fluid. The patient was stabilized with antiviral treatment, methylprednisolone, plasmapheresis and cycophosphamide. Natalizumab was discontinued. This case illustrates the neuroimmunological and neuroinfectiological consequences of treatments with biologicals that influence the immune system.

## Background

Natalizumab is a humanized monoclonal antibody directed against the alpha4 subunit of integrins that are expressed on activated T cells and other leukocytes. Blocking the interaction between T cells and endothelial vascular cell adhesion molecule 1 (VCAM-1) inhibits transmigration of T cells through the blood brain barrier into the central nervous system (CNS) [1,2]. Based on large clinical trials [3,4] natalizumab was approved for the treatment of relapsing-remitting multiple sclerosis (RRMS). However, due to the occurrence of progressive multifocal leukencephalopathy (PML) under natalizumab treatment [5], its use is recommended only in selected patients that have either failed to respond to other disease-modifying treatments or show a very active disease. Other infections are not considered to be of major concern in patients treated with natalizumab.

## Case presentation

The 49-year-old patient was diagnosed with relapsing-remitting multiple sclerosis (RRMS) in 2006 that was initially treated with interferon beta-1a. Due to continuous relapse activity, the therapy was escalated to monthly natalizumab infusions in 2007. Thereafter, he had been stable with only one relapse in the first year of natalizumab treatment. In December 2012, he presented in our neurological emergency department with a progressive bilateral visual loss and slight confusion lasting for two weeks. Prior to admission he had been treated in a neurological practice with high dose methylprednisolone due to suspected relapse.

**Figure 1:**
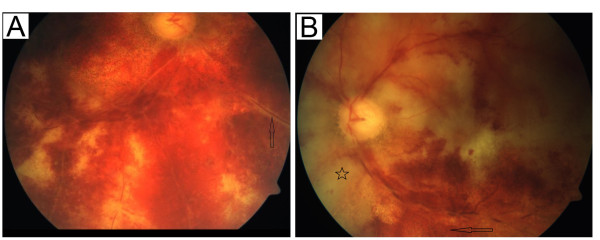
**Fundoscopic examination at the beginning and after treatment.** The initial fundoscopic examination of the right eye **(A)** shows a peripheral necrotizing retinitis and periarteritis (arrow). The follow-up examination after treatment **(B)** of the left eye presents an extensive posterior retinal necrosis (pentangle) and pigmentary changes of affected areas (arrow).

**Figure 2:**
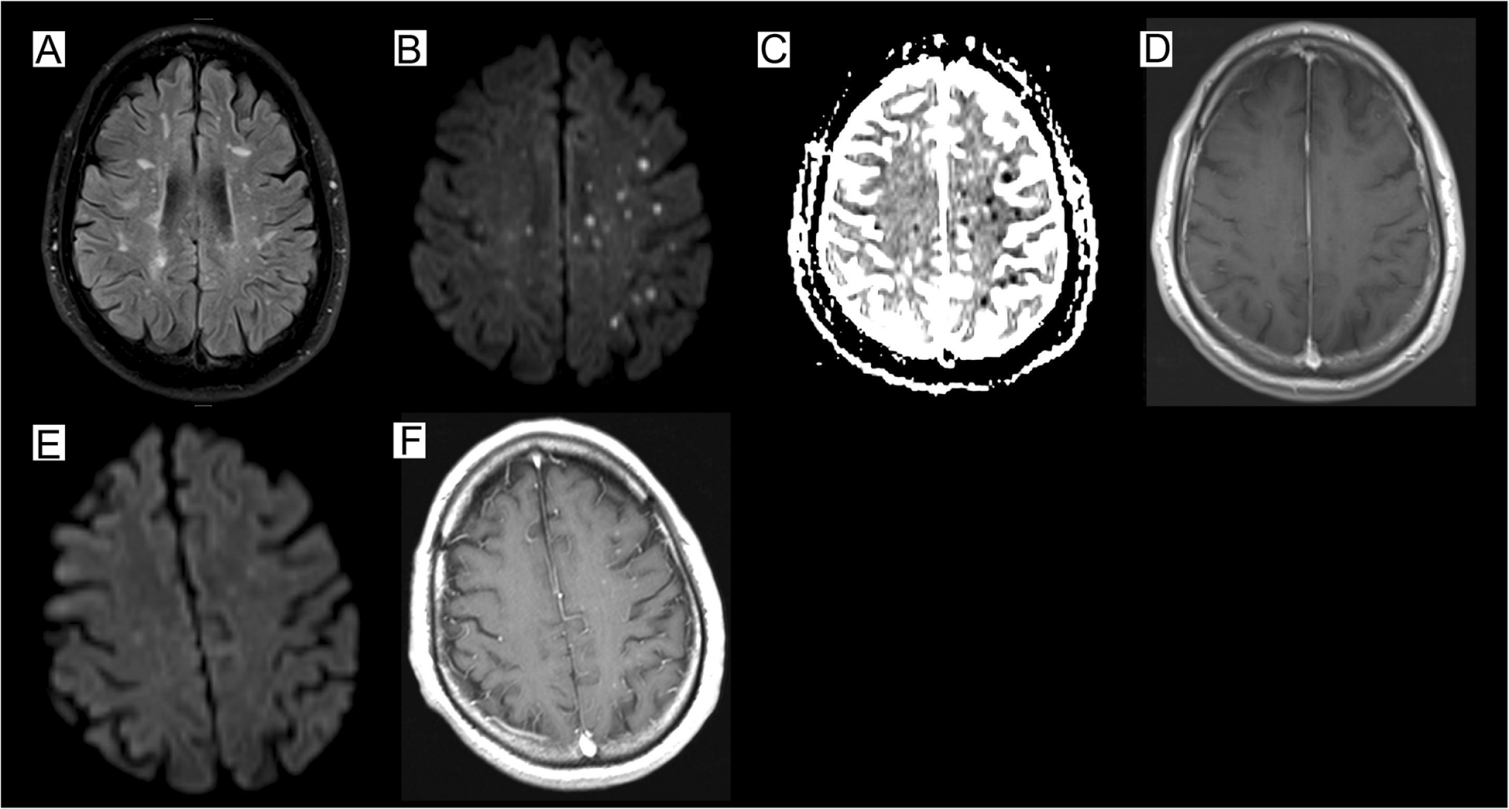
**Brain magnetic resonance imaging (MRI) at the initial presentation and after two months.** Brain MRI five days after admission **(A-D)** shows a FLAIR-weighted image with non-active MS typical lesions **(B)**, a diffusion-weighted image illustrating multiple dot-shaped cortical and subcortical lesions with restricted diffusion not limited to a large-vessel vascular territory and corresponding reduction on the apparent diffusion coefficient sequence **(B,C)**, a contrast enhanced T1-weighted image without any intracerebral contrast enhancement (**D**; circular contrast uptake of the cerebral meninges due to a previous lumbar puncture). The follow-up MRI after two months **(E-F)** shows a diffusion-weighted image without any new lesions **(E)** and a contrast enhanced T1-weighted image with dot-shaped contrast-enhancing lesions **(F)** in contrast to the initial presentation.

On neurological examination, he demonstrated a previously described residual left-sided hemiparesis, pronounced in the left leg with brisk reflexes, an amaurosis of his right eye and markedly reduced vision of his left eye. The ophthalmological examination of the anterior segment of both eyes showed no inflammation. The posterior segment revealed an occlusive periarteriitis and a patchy necrotizing retinitis (Figure 1A-B). Besides the known non-active multiple sclerosis (MS)-typical lesions, the cranial magnetic resonance imaging (MRI) displayed multiple new dot-shaped cortical and subcortical lesions with diffusion restrictions that were not limited to a large-vessel vascular territory (Figure 2A-D). Neither these lesions nor the basal vessels revealed any contrast enhancement.

Cerebrospinal fluid (CSF) analysis demonstrated a normal cell count, positive oligoclonal bands in the CSF (type 2), a total protein elevation of 0.87 g/L, an albumin quotient (CSF/serum) of 12.3 and varicella zoster virus (VZV) DNA was tested positive by PCR amplification. All other virological and microbiological CSF investigations were negative (antibody indices for CMV/HSV/VZV, CMV pp65 antigen, EBV antibodies and PCR, JCV PCR, HIV antigen ELISA, tuberculosis culture and PCR, aspergillus antigen ELISA, leptospirosis antibodies, cryptococcus antigen immunodiffusion, toxoplasmosis antibodies, borrelia antibodies, culture for neisseria and brucella and an unspecific culture for other pathogenic and non-pathogenic bacteria). Transesophageal echocardiography, electrocardiography, Doppler/duplex sonography of the deep leg veins and carotid arteries, coagulopathy screening, autoimmune antibodies (anti-neutrophil cytoplasmic antibody, anti-nuclear antibodies, ENA antibodies, anti-DNA antibodies, anti-NMDAR antibodies, anti-AMPAR antibodies, anti-LG1 antibodies, anti-CASPR2 antibodies, anti-GABAR antibodies, anti-phospholipid antibodies, anti-transglutaminase antibodies and anti-gliadin antibodies) and blood sedimentation rate were normal.

As we suspected a vasculitis based on the fundoscopic examination and the MRI, we performed a digital subtraction angiography and a MRI-guided diagnostic brain biopsy of the parenchyma and the meninges. Both were carried out during the initial immunosuppressive and antiviral treatment due to the rapid progression of the visual loss. Neither the conventional angiography nor the biopsy showed definite signs of a vasculitis; however, the biopsy did not include deep white matter.

For treatment of the VZV infection, aciclovir (800 mg three times a day iv for 14 days) was initiated. We also applied a high dose of intravenous methylprednisolone (1 g/day for five days followed by 2 g/day for five days) and subsequently an oral steroid (prednisolone 80 mg/day). Moreover, five cycles of plasma exchange therapy were performed to accelerate natalizumab clearance. After the steroid treatment and plasma exchange therapy, we applied a bolus of cyclophosphamide (500 mg/m^2^). In parallel, we treated the patient with acetylsalicylic acid (100 mg/day) and simvastatin (20 mg/day). Before transferral to the neurological rehabilitation clinic, he was almost completely blind and reported typical signs of a Charles-Bonnet syndrome with complex vivid hallucinations.

At follow-up after two months, the CSF of the clinically stable patient showed a more than six-fold increase of intrathecal antibody indices against VZV from 0.900 at first presentation to 5.900, while the viral PCR results, including John Cunningham virus (JCV) PCR, were negative. The follow-up MRI after two and four months did not reveal any diffusion restrictions, whereas the dot-shaped lesions were persistently contrast-enhancing in contrast to the initial presentation. This was interpreted as an ongoing immune response related to the apparently still active vasculitis (Figure 2E-F). Therefore, the patient further received cyclophosphamide on a monthly basis. Since we assume that the infection associated vasculitis is a transient phenomenon and the active MS requires further long term treatment, it is planned to switch the treatment to teriflunomide.

## Conclusions

In analogy to a natalizumab-associated PML with a reactivation of JCV, there might be a risk for new infections or reactivation of other CNS viral infections due to a decline of CD4 T cells under treatment with natalizumab. Several cases of herpes simplex virus (HSV) encephalitis and meningitis in patients on natalizumab [6-8] and herpes zoster due to VZV reactivation have been reported [9]. In a recently published case series four cases of VZV reactivation with meningitis, meningomyelitis and meningoradiculitis were noted, but none with a presentation of vasculitis [6].

Even though reactivations of HSV and VZV under natalizumab have already been described, this is the first report of a combined retinal and CNS vasculitis associated with a VZV reactivation in a patient treated with natalizumab. VZV is the only virus that is able to replicate within arteries [10] and can consequently lead to an inflammatory vasculopathy by invasion of small and large arteries of the brain [11,12]. The vasculopathy can present with various neurological manifestations such as stroke, neuropathy or encephalitis.

The pronounced ophthalmological findings of an occlusive periarteriitis and multifocal patches of necrotizing retinitis were typical manifestations of an infection-associated vasculitis and the progression despite intensive immunosuppressive treatment argue against a bilateral optic neuritis as a relapse of MS. Furthermore, MRI lesions were located cortically as well as subcortically and exhibited diffusion restrictions initially and prolonged contrast enhancements two to four months later. High dose steroid therapy could have been the reason why there was no contrast uptake of the lesions at first. The negative result of the angiography does not argue strongly against a vasculitis, considering that in 30% of the VZV vasculitis cases the angiography can be negative [13] and that the angiography was performed under immunosuppression. Moreover, the small dot-shaped lesions in the MRI suggest an affection of the small vessels, which cannot be detected by conventional angiography. The biopsy was probably performed too superficially and also during immunosuppression, leading to a negative result.

Another unusual aspect of this case is the normal CSF cell number despite positive PCR for VZV. It may be speculated that due to the natalizumab treatment the cell number in the CSF cannot adequately rise. It is well described that the cell number in the CSF is decreased and the CD4/CD8 ratio altered upon natalizumab treatment [14]. Thus, one has to be even more vigilant for such an infection since the typical CSF cell numbers maybe misdirecting.

It is challenging to differentiate CNS infections and associated vasculitides under monoclonal antibody treatment from an MS relapse. The case illustrates that biologicals have neuroimmunological and neuroinfectiological consequences and both of them need to be considered at the same time. Therefore, neurologists should pay special attention to possible viral CNS infections when prescribing natalizumab to start an adequate and early treatment. In any case, it is necessary to rule out a PML, which is a serious complication of natalizumab treatment [15]. Possible future challenges are to define high-risk patients for viral reactivations and to evaluate the role of oral antivirals for prevention.

## Consent

Written informed consent was obtained from this patient for the publication of the case report and any accompanying images. A copy of the written consent is available for review by the Editor-in-Chief of this journal.

## Abbreviations

AMPAR: α-amino-3-hydroxy-5-methyl-4-isoxazoleproprionic acid receptor; CASPR2: contactin-associated protein-like 2; CMV: cytomegalovirus; CNS: central nervous system; CSF: cerebrospinal fluid; DNA: deoxyribonucleic acid; EBV: Epstein-Barr virus; ELISA: Enzyme Linked Immunosorbent Assay; ENA: extractable nuclear antigen; GABAR: γ-aminobutyric acid receptor-B; HIV: human immunodeficiency virus; HSV: herpes simplex virus; JCV: John Cunningham virus; LG1: leucine-rich glioma inactivated protein 1; MRI: magnetic resonance imaging; MS: multiple sclerosis; NMDAR: N-methyl-D-aspartate receptor; PCR: polymerase chain reaction; PML: progressive multifocal leukencephalopathy; VCAM-1: endothelial vascular cell adhesion molecule 1; VZV: varicella zoster virus.

## Competing interests

Dr. Stangel reports grants and personal fees from Bayer Healthcare, personal fees from Baxter, grants and personal fees from Biogen Idec, personal fees from CSL Behring, personal fees from Grifols, grants and personal fees from Novartis, personal fees from Sanofi-Aventis, grants and personal fees from Teva, outside the submitted work. All other authors report no conflicts of interest.

## Authors’ contributions

XK has acquired, analyzed and interpreted the clinical data and drafted the manuscript. FW and MS have made contributions to the analysis and interpretation of the data and have been involved in drafting the manuscript. IB has performed and analyzed the fundoscopic examinations. MD carried out the MRI examinations and interpreted them. RD has analyzed the data and revised the manuscript critically for intellectual content. All authors have given final approval of the version to be published.
